# ToxGen: an improved reference database for the identification of type B-trichothecene genotypes in *Fusarium*

**DOI:** 10.7717/peerj.2992

**Published:** 2017-02-15

**Authors:** Tomasz Kulik, Kessy Abarenkov, Maciej Buśko, Katarzyna Bilska, Anne D. van Diepeningen, Anna Ostrowska-Kołodziejczak, Katarzyna Krawczyk, Balázs Brankovics, Sebastian Stenglein, Jakub Sawicki, Juliusz Perkowski

**Affiliations:** 1 Department of Botany and Nature Protection, University of Warmia and Mazury, Olsztyn, Poland; 2 Natural History Museum, University of Tartu, Tartu, Estonia; 3 Department of Chemistry, Poznań University of Life Sciences, Poznań, Poland; 4 CBS-KNAW Fungal Biodiversity Centre, Utrecht, Netherlands; 5 Institute of Biodiversity and Ecosystem Dynamics, University of Amsterdam, Amsterdam, Netherlands; 6 Laboratorio de Biología Funcional y Biotecnología (BIOLAB)-CICBA-INBIOTEC, CONICET, Azul, Buenos Aires, Argentina; 7 Cátedra de Microbiología-Facultad de Agronomía de Azul-UNCPBA, Azul, Buenos Aires, Argentina; 8 Department of Biology and Ecology, University of Ostrava, Ostrava, Czech Republic

**Keywords:** Trichothecene genotypes, *Fusarium*, Chemotypes, Molecular identification, Annotation

## Abstract

Type B trichothecenes, which pose a serious hazard to consumer health, occur worldwide in grains. These mycotoxins are produced mainly by three different trichothecene genotypes/chemotypes: 3ADON (3-acetyldeoxynivalenol), 15ADON (15-acetyldeoxynivalenol) and NIV (nivalenol), named after these three major mycotoxin compounds. Correct identification of these genotypes is elementary for all studies relating to population surveys, fungal ecology and mycotoxicology. Trichothecene producers exhibit enormous strain-dependent chemical diversity, which may result in variation in levels of the genotype’s determining toxin and in the production of low to high amounts of atypical compounds. New high-throughput DNA-sequencing technologies promise to boost the diagnostics of mycotoxin genotypes. However, this requires a reference database containing a satisfactory taxonomic sampling of sequences showing high correlation to actually produced chemotypes. We believe that one of the most pressing current challenges of such a database is the linking of molecular identification with chemical diversity of the strains, as well as other metadata. In this study, we use the Tri12 gene involved in mycotoxin biosynthesis for identification of Tri genotypes through sequence comparison. Tri12 sequences from a range of geographically diverse fungal strains comprising 22 *Fusarium* species were stored in the ToxGen database, which covers descriptive and up-to-date annotations such as indication on Tri genotype and chemotype of the strains, chemical diversity, information on trichothecene-inducing host, substrate or media, geographical locality, and most recent taxonomic affiliations. The present initiative bridges the gap between the demands of comprehensive studies on trichothecene producers and the existing nucleotide sequence databases, which lack toxicological and other auxiliary data. We invite researchers working in the fields of fungal taxonomy, epidemiology and mycotoxicology to join the freely available annotation effort.

## Introduction

Trichothecenes are one of the largest and the most studied group of mycotoxins. These non-volatile sesquiterpene epoxide compounds can induce mycotoxicoses in humans and domestic animals, and might play a role in the virulence of individual fungal strains (Desjardins, 2006; Marin et al., 2013). Trichothecenes can be classified into four groups (Types A, B, C, and D) based on the substitution pattern of tricyclic 12,13-epoxytrichothec-9-ene (EPT) (McCormick et al., 2011). The most frequently reported in wheat- and barley-growing regions are type B trichothecenes including deoxynivalenol (DON), nivalenol (NIV), and their acetylated derivatives: 3-acetyldeoxynivalenol (3ADON), 15- acetyldeoxynivalenol (15ADON), and 4-acetylnivalenol (4ANIV, syn. fusarenone-X) (Desjardins, 2006; Marin et al., 2013).

In general, contamination of grain with these mycotoxins is attributed to the panglobal distribution of *Fusarium graminearum* sensu stricto (s.s.), which is the major cause of Fusarium head blight (FHB), a devastating disease of small grain cereals (Van der Lee et al., 2015). *Fusarium graminearum* sensu stricto (s.s.) belongs to the monophyletic fungal complex referred to as *F. graminearum* species complex (FGSC), which consists of at least 16 phylogenetic species (Sarver et al., 2011). In Europe, *F. graminearum* s.s. has displaced *F. culmorum*, which had been the major FHB agent of wheat since the 1840s (Van der Lee et al., 2015). However, several recent surveys identified the predominance of *F. culmorum* in different European localities, suggesting the role of climatic conditions in driving the prevalence of each species (Scherm et al., 2013). Both *F. culmorum* and *F. graminearum* s.s., as well as other members of the FGSC, can belong to different chemotypes: 3ADON (producing DON and 3ADON), 15ADON (producing DON and 15ADON) (absent in *F. culmorum*) and NIV (producing NIV and 2ANIV) (Ward et al., 2002). It is worth noting, however, that strains assigned to particular chemotypes can co-produce minor amounts of compounds characteristic for other chemotypes in inoculated grain or solid media (Mugrabi de Kuppler et al., 2011; Pasquali et al., 2016a; Pasquali et al., 2016b), which can not be ignored in terms of food safety. A multiple mycotoxin-contaminated diet may cause more serious damage than a single mycotoxin one (Hou et al., 2013).

Identification of chemotypes within *Fusarium* field populations appears to be important in determining population structure, monitor chemotype changes and predict the presence of particular trichothecene molecules in plant material (Mugrabi de Kuppler et al., 2011; Pasquali et al., 2010; Zhang et al., 2010).

Chemotypes are usually identified using a chemical method, after the toxin detected in the media at highest amount. However, media and incubation conditions have a very strong influence on mycotoxin production (Pasquali & Migheli, 2014; Buśko et al., 2014; Frisvad, 2012). For example, *F. graminearum* s.s. produces limited quantities of trichothecenes in liquid media (Kimura et al., 2003), favouring the production of acetyl derivatives of DON rather than DON (Pestka, el-Bahrawy & Hart, 1985). In contrast, grain infected with DON chemotypes is predominantly contaminated with DON and with smaller amounts of 3ADON or 15ADON (Audenaert et al., 2013). There is no consensus on which media are optimal for metabolite production, but a solid agar medium is recommended for the exploration of chemical diversity (Frisvad, 2012).

Beside external factors, the translation of genotype into chemotype is complicated by gene interactions leading to high strain-dependent mycotoxin diversity (Desjardins et al., 2008), often reported as significant variation in amounts of toxin production (Pasquali et al., 2016a; Pasquali et al., 2016b), simultaneous production of minor amounts of molecules specific for other chemotypes (Mugrabi de Kuppler et al., 2011; Pasquali et al., 2016a; Pasquali et al., 2016b), and even production of high (or highest) quantities of trichothecene compounds not characteristic for a certain Tri genotype (Castañares et al., 2014; Kulik et al., 2016).

Recently, the sequencing of genes responsible for trichothecene production (Tri genes) allowed for determination of the genetic basis of chemotypespecific differences within type B trichothecene producers (Ward et al., 2002). Subsequently, a number PCR-based assays have been developed for quick chemotype prediction. These molecular methods are mainly based on the use of specific sets of primers and/or probes (Lee et al., 2001; Ward et al., 2008; Kulik, 2011; Nielsen et al., 2012; Pasquali & Migheli, 2014). However, specific molecular assays are prone to false-positive and -negative results (Desjardins et al., 2008; Wang et al., 2008), most likely due to unexpected mutations in the primer binding sites or large deletion/insertion polymorphisms which result in amplification biases.

Next generation sequencing methods (NGS) can offer more detailed, high-throughput identification of trichothecene (Tri) genotypes in large sample sets as compared to previous molecular methods in terms of detail and magnitude. NGS instruments generate millions of reads per run and are increasingly seen as one of the primary information sources for species identification in many groups of organisms, including fungi (Lindahl et al., 2013; Shokralla et al., 2014). The procedure usually involves the sequencing of NGS libraries, assembly, and comparison with databases with annotated entries (Nilsson et al., 2006; Begerow et al., 2010). We state that one of the most pressing current challenges of such a database is the linking of molecular identification with other traits, e.g., mycotoxin production. In the present paper, we demonstrate the usefulness of the Tri12 gene involved in mycotoxin biosynthesis for the identification of Tri genotypes through sequence comparison. We analyzed the GenBank database to check: (1) if this database contains a satisfactory taxonomic sampling of Tri12 sequences from a range of species known to produce type B trichothecenes, (2) if the sequences in the reference database are correctly identified to the Tri genotype/chemotype and species level and feature informative annotation.

Tri12 orthologues proved to be missing from approximately 40% of the members of FGSC. Hence, we sequenced Tri12 orthologues from a set of reference strains of *F. brasilicum*, *F. gerlachii*, *F. louisianense*, *F. nepalense*, *F. ussurianum, F. vorosii* and the strain CBS 123663 (NRRL 344461) (this strain lacks a species name), as well as one strain of *F. culmorum*. We found that the GenBank database does not provide informatively annotated entries facilitating proper identification of Tri genotypes.

To fill this gap we created a novel curated database combining data on both genotype and actual mycotoxin production, and we invite fellow researchers to use the database and join in its further annotation efforts.

## Materials & Methods

### Fungal strains and growth conditions

Seven fungal strains of the *F. graminearum* species complex were subjected to next generation sequencing analysis: CBS 119180 (*F. brasilicum*), CBS 123666 (*F. gerlachii*), CBS 127524 (*F. louisianense*), CBS 127503 (*F. nepalense*), CBS 123754 (*F. ussurianum*), CBS 123664 (*F. vorosii*), and CBS 123663 (lacking a formal species name). In addition, one *F. culmorum* strain (CBS 110262) was incorporated into the analysis.

All strains have been maintained in international fungal collections: CBS - Fungal Biodiversity Centre, Utrecht, the Netherlands, and the ARS Culture Collection, USDA, Peoria, IL, US. A detailed description of the fungal strains is given in the ToxGen database. Six-to-eight-week-old laboratory stock cultures were maintained at 4 °C on PDA slants for plate inoculation. For DNA extraction, fungal strains were incubated on Petri plates (Ø80 mm) with PDA medium at 24 °C in the dark for 7 days.

### DNA isolation

DNA was extracted from 0.1 g of mycelium scraped from the surface of PDA plates as previously described in Kulik et al. (2016).

### DNA sequencing, assembly and annotation of Tri12 sequences

Whole genome libraries were prepared using the Nextera XT kit (Illumina, San Diego, CA, USA) from genomic DNA as previously described in Kulik et al. (2016).

### Assembly and annotation of Tri12 sequences

For assembling, de-multiplexed and trimmed reads were aligned to the complete sequence of the Tri12 gene of F. graminearum s.s. strain GZ3639, from which the complete core Tri cluster is deposited in the GenBank database under accession number AF359361. Sequence reads were aligned with Geneious (v.6.1.6 created by Biomatters, available from http://www.geneious.com) as previously described in Kulik et al. (2016). Annotations were performed using Geneious software based on Tri sequence data deposited under accession numbers: AF359361 and KP057243. Complete Tri12 sequences have been deposited in the NCBI database under the GenBank accession numbers: KX197185, KX197186, KX197187, KX197188, KX197189, KX197190 and KX197191. Tri sequence data of CBS 110262 have been deposited in the NCBI database under the GenBank accession number KU572424.

### Downloading Tri12 sequences with associated metadata from GenBank

Forty-seven sequences of Tri12 genes (around 1920 nt) were downloaded with the Geneious software from the GenBank database. In addition, Geneious enabled access to sequence information stored in the GenBank database, which included: unique strain numbers, taxonomic affiliations and publication data.

### Determination of sequence similarity thresholds (%) within and among Tri12 genotypes

Sequences of the Tri12 gene (1,915 nt) from 65 fungal strains were aligned with ClustalW genome alignment within the Geneious environment. In order to calculate similarity thresholds (%) within and among Tri genotypes, all sequences were compared to each other for similarity. Percentages of nucleotide similarities of all possible pairwise combinations were determined.

### Analyses of pairwise distances

Genetic distance over sequence pairs within and among genotypes was calculated with MEGA v.6 software (Tamura et al., 2013), based on the Kimura 2-parameter model (K2P) of nucleotide substitution (Kimura, 1980). The variation rate among sites was modelled with a gamma distribution (shape parameter =0.4). The best-fit model of DNA sequence evolution was chosen using MEGA v.6. After calculating the genetic distance matrix for all sequence pairs, the number of each resulting value was counted within and among genotypes and shown on the graph.

### Evaluation of barcode effectiveness

Sequence similarity was also verified by direct comparison of DNA sequences in the Species Identifier v.1.7.7 from TAXONDNA software package (Meier et al., 2006). The analyzed 65 sequences were *a priori* assigned to 3ADON, 15ADON or NIV genotype. Then two methods: ‘best match’ and ‘best close match’ were applied to test whether particular sequences allow for the correct identification of the genotype. The program compared each successive sequence with all the other sequences present in our data set and matched them with the most similar ones. If the indicated pair came from one genotype, then in the ‘best match’ analysis the result was considered positive. If the sequences did not belong to the same genotype, the result was referred to as ‘incorrect.’ Several equally good best matches from different groups were considered ‘ambiguous.’ In the ‘best close match’ method the threshold was computed from pairwise distances. All the results above the threshold were classified as ‘no match.’ The analyses were carried out with the use of K2P corrected distances and a minimum sequence overlap of 250 bp.

### Tree based assessment

A tree-based method was used to assess whether sequences in our data set form genotype-specific clusters. The maximum likelihood (ML) method based on the K2P model and uniform rates among sites was done with MEGA v.6. The tree was tested with the bootstrap method (Felsenstein, 1985) using 1,000 replicates.

### Accessing information on fungal strains, storage and annotation of sequence data in the ToxGen database

Historical data on the fungal strains were gathered from peer-reviewed articles, as well as from the Fungal Biodiversity Centre strain database (CBS) (http://www.cbs.knaw.nl). PlutoF (https://plutof.ut.ee/) platform enables the rapid submission, retrieval, and analysis of study, specimen and various sequence data. Its ultimate goal is to cover all elements of the extant biodiversity, and ecological, genetic and taxonomic diversity (Abarenkov et al., 2010). A total of 65 sequences of the Tri12 gene were stored in PlutoF. Each deposited sequence in the database was linked to gathered metadata, which included all available strain numbers indicating culture collections maintaining the strains, most recent taxonomic affiliations, indication on Tri genotype (either 3ADON, 15ADON or NIV), indication on chemotype (either 3ADON, 15ADON, NIV or unknown), details on mycotoxin production including type of trichothecene inducing host, substrate or medium, isolation source, geographical locality and literature references. Species names of the strains were checked for validity, legitimacy and linguistic correctness in the MycoBank database owned by the International Mycological Association (http://www.mycobank.org).

## Results

### DNA sequencing, assembly and annotation of Tri sequence data

In this study we sequenced, assembled and annotated Tri12 sequences from strains representing seven phylogenetic species within FGSC, sequences of which were not available in the GenBank database: CBS 119180 (*F. brasilicum*), CBS 123666 (*F. gerlachii*), CBS 127524 (*F. louisianense*), CBS 127503 (*F. nepalense*), CBS 123754 (*F. ussurianum*), CBS 123664 (*F. vorosii*), and the strain CBS 123663 (NRRL 344461). In addition, the conflicting metadata of the strain CBS 110262 (*F. culmorum*) reported in the literature prompted us to include this strain in the analysis.

### Determination of sequence similarity thresholds (%) within and among Tri12 genotypes

**Figure 1:**
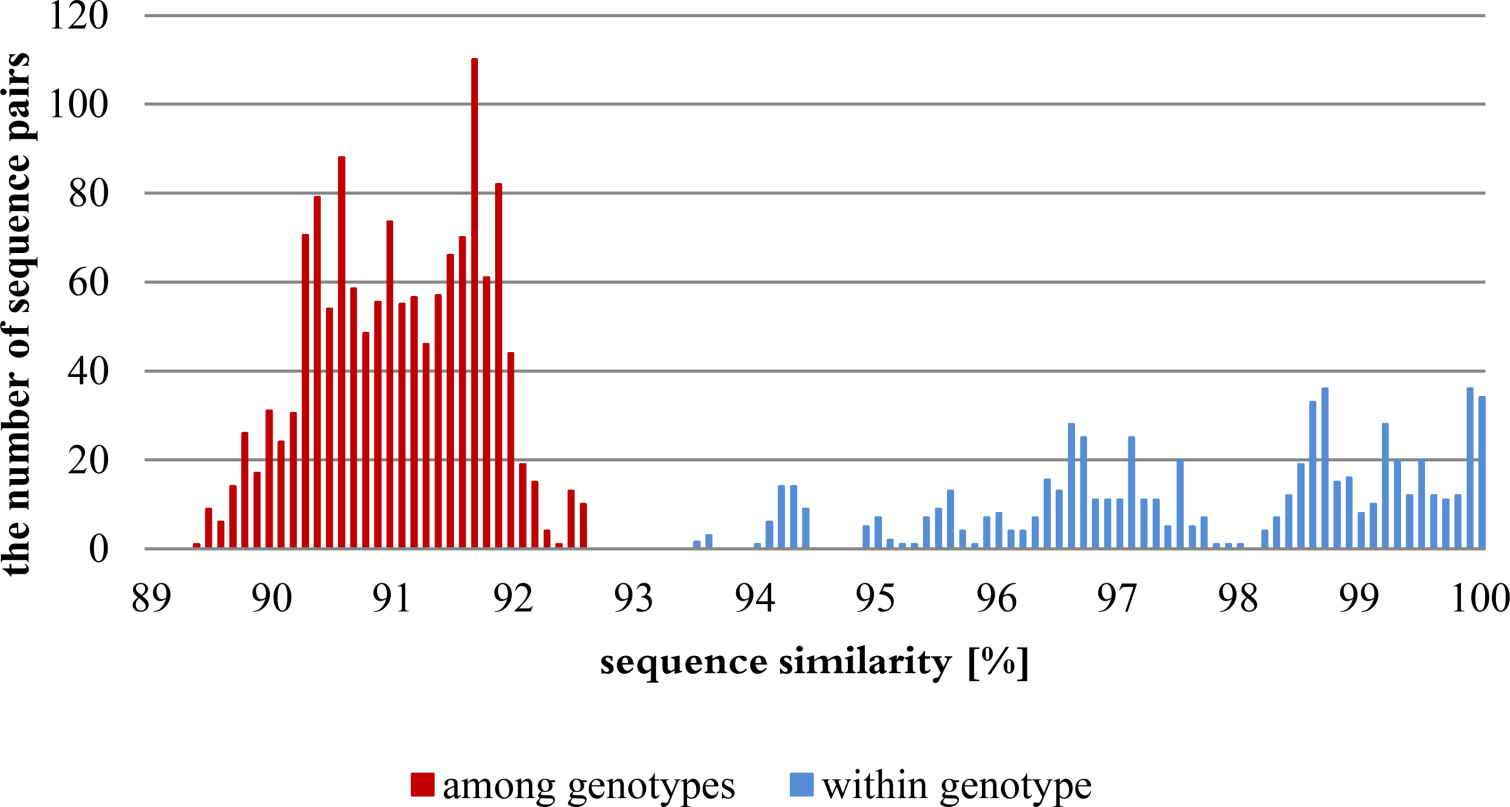
Distribution of genetic variation within and among genotypes based on percentages of nucleotide similarities.

The highest intra-genotype variations were 6.5%, 3.6% and 4.1%, for 3-, 15ADON and NIV genotypes, respectively. The inter-genotype variations were 7.4–10.3%, 8.4–10.6% and 7.8–9% between 3ADON and 15ADON, 3ADON and NIV, and 15ADON and NIV genotypes, respectively. The threshold similarity values between 3ADON and 15ADON, 3ADON and NIV, and 15ADON and NIV genotypes were 0.9%, 1.9% and 3.7%, respectively. The results are shown on the graph (Fig. 1).

### Analyses of pairwise distances

**Figure 2:**
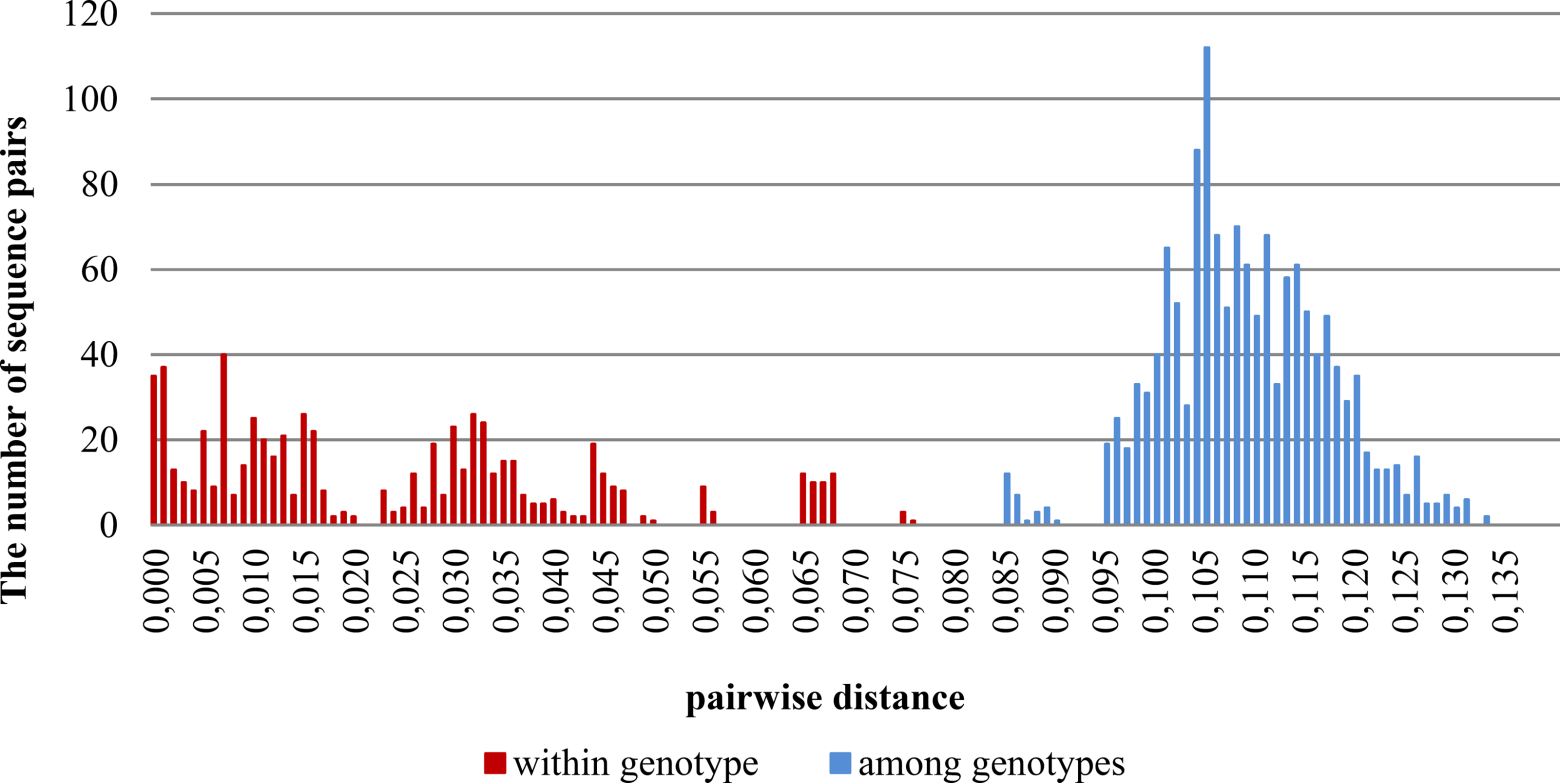
Distribution of pairwise distances within and among genotypes based on the Kimura 2-parameter model of nucleotide substitution.

The analysis of genetic distance between Tri12 sequence pairs shows no overlap between distance values within and among genotypes, and hence indicates the existence of a so-called barcoding gap (Kress & Erickson, 2007). The results are shown on the graph (Fig. 2).

### Evaluation of barcode effectiveness

The analysis performed with the ‘Best match’ method showed 100% efficiency of the analyzed DNA fragments in genotype identification (Table 1). The ‘best close match’ method allowed for 100% correct genotype discrimination at a 3.1% threshold.

**Table 1:** Identification success of three Tri genotypes based on Tri12 gene using Species Identifier 1.7.7 software under ‘best match’ and ‘best close match’ methods.

**Best match (%)**			**Best close match (%)**				Threshold
Correct	Ambiguous	Incorrect	Correct	Ambiguous	Incorrect	No match	
**100**	0	0	**100**	0	0	0	3.1

### Tree based assessment

**Figure 3:**
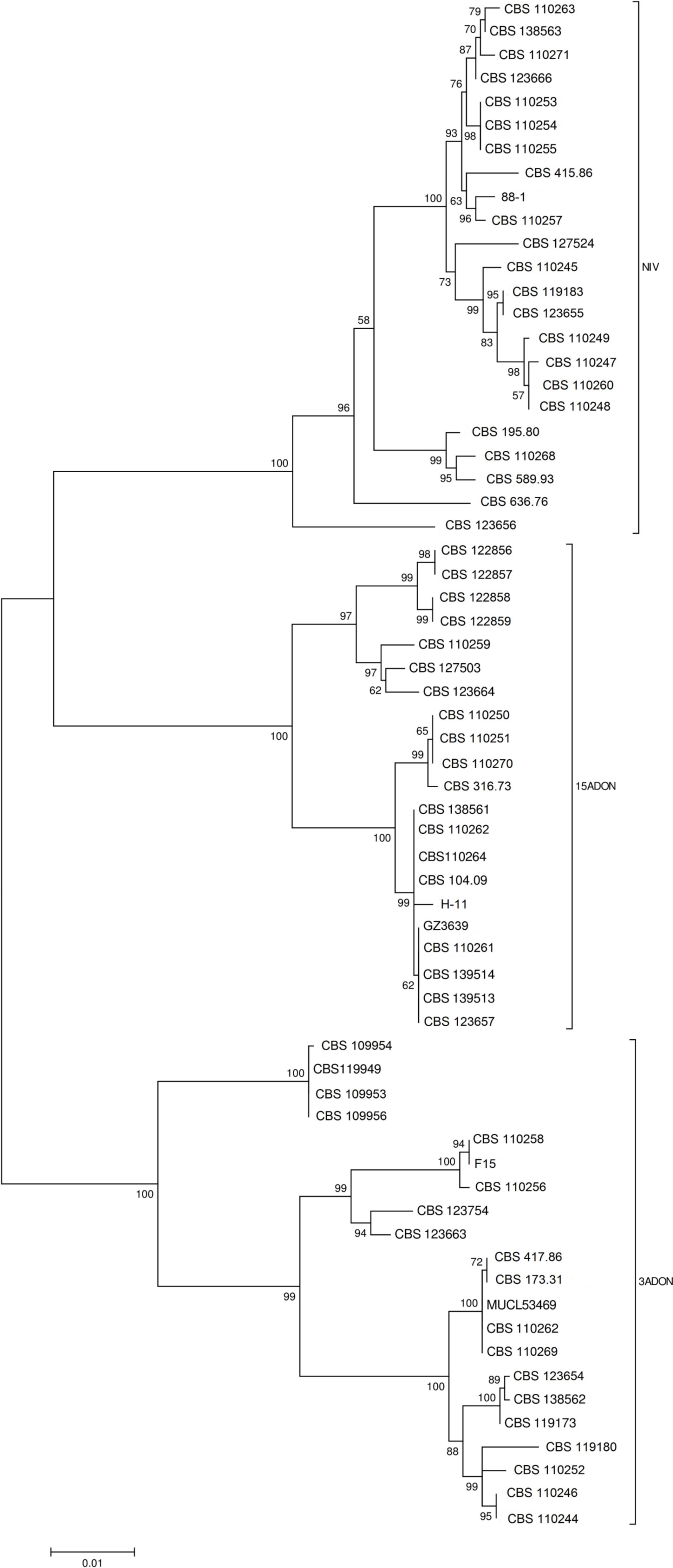
Phylogenetic mid-point rooted tree generated by maximum likelihood (ML) trees from 63 sequences of Tri12 gene, comprising 1,915 characters. Numbers on the branches indicate percentages of 1,000 bootstrap-replications, with 70% as cut-off.

Tree-based DNA identification of genotypes was assessed using the Maximum Likelihood (ML) method without considering an outgroup. As shown in Fig. 3, ML analysis delineated Tri genotypes into three well supported clades with bootstrap values of 100%.

### Examination of the Tri12 sequence data from the GenBank database underlines the need for a new reference database for the identification of Tri genotypes

The rapidly accumulating DNA sequence data stored in the International Nucleotide Sequence Databases (INSD) (GenBank, EMBL, and DDBJ) are vital to studies in mycology, including fungal ecology, epidemiology, biogeography and taxonomy (Nilsson et al., 2006). Molecular mycology relies to a great extent not only on the sequence data itself, but also on the associated metadata, which includes information on, e.g., geographic origin, host or substrate of isolation, fungicide resistance, and in the case of toxigenic fungi—mycotoxin production. Unfortunately, there is no requirement to submit metadata alongside the sequences, which results in the well known poor annotation of sequence data stored in INSD (Abarenkov et al., 2010). In this study, we found that the Tri12 sequences stored in the GenBank database are poorly supplied with metadata, making it an unsuitable source of information for more comprehensive studies. Genbank entries do not provide chemotaxonomic information which could enable direct determination of Tri genotypes of the fungal strains, and lacks other auxiliary information of the strains, such as mycotoxin profiles, collection site, origin, etc. Most (all but one) entries in GenBank contain unique strain numbers assigned originally by internationally recognized culture collections, which were further used by us to search for all possible metadata regarding particular strains using a web browser.

It is worth noting that the same fungal strains are often kept and distributed by different culture collections which use their own unique strain codes. This results in the use of multiple strain codes in peer-reviewed papers, which makes strain recognition problematic. This situation is also reflected in the GenBank database. We found that multiple entries for single strains are often annotated with different strain identifiers and without an indication of their origin. An example illustrating the above situation can be given on the basis of a single strain whose multiple sequence data (a total of 24 GenBank records) has been annotated in GenBank with two different strain codes: GZ3639 and NRRL 29169. Notably, GenBank contains two overlapping core Tri sequences of this strain: AF359361 (57,840 bp) and AY102599 (18,533 bp), whose differential annotation makes quick recognition difficult.

As previously indicated in Geiser et al. (2013), the application of *Fusarium* nomenclature in GenBank is also inconsistent. We found that all entries annotated as *F. graminearum* were consistently converted to their teleomorph name *Gibberella zeae*, which should be not used due to changes in the International Code of Nomenclature for algae, fungi, and plants (Geiser et al., 2013). None of the Genbank entries included the name *F. graminearum* sensu stricto, which describes a phylogenetic species being the major causal agent of FHB in wheat and barley in Europe, the United States, South America and portions of Asia (Gale et al., 2007). Although widely used in numerous peer-reviewed articles, the above species name is still neither documented in MycoBank database nor used in strain descriptions of culture collections.

In this study, we checked species names consistency between GenBank and culture collections’ strain descriptions. We found that for all but one strain the metadata found in GenBank had species names consistent with data found in both CBS and ARS culture collections. The exception was conflicting metadata of the strain isolated from millet in Hungary. GenBank metadata on this strain (NRRL 6394) is consistent with ARS Culture Collection (NRRL) strain data, which assigned this strain as *F. graminearum*, but in contrast to the CBS strain database (CBS 110262), where this strain was assigned as *F. culmorum.* To confirm its species identity, we recovered translation elongation 1-alpha (TEF1) from short reads obtained by genome sequencing. A BLASTN search of the TEF1 sequence of CBS 110262 (KT008433) showed 100% similarity to *F. culmorum* (KP008988). Independent sequencing of TEF1 of CBS 110262 performed at CBS also confirmed the taxonomic status of CBS 110262 as *F. culmorum*. Thus, it seems that the CBS and NRRL culture collections maintain two different strains which should never be considered as equivalents.

### Creation of a new reference database for the identification of Tri12 genotypes

The identification of microorganisms through sequence comparison largely depends on the development of high quality sequence databases that are thoroughly curated by taxonomists and systematists (Begerow et al., 2010). In this study, the reference database consisting of Tri12 sequence data was built on PlutoF. The main purpose of creating such a database is to provide a joint corpus of molecular and toxicological metadata. Our database now contains sequences from 65 fungal strains covering 22 fungal species within a group of type B trichothecene producers. It includes strains representing all currently known phylogenetic species within FGSC (Aoki et al., 2012), and other species considered among the most relevant type B trichothecene producers: *F. cerealis*, *F. culmorum*, *F. dactylidis*, *F. lunulosporum*, and *F. pseudograminearum*. Production of a type B trichothecene (nivalenol) has also been reported by strains of *F. poae* (Jestoi et al., 2008) and *F. equiseti* (Kosiak et al., 2005). However, due to their relatively low trichothecene production they have not yet been considered in our study. Our reference database has implemented a total of 519 annotations, taking into account specifications of: all available strain numbers indicating culture collections maintaining the strains, most recent taxonomic and chemotaxonomic affiliations, information on trichothecene production by the strains, including type of medium, substrate or host inducing mycotoxin production, specifications of geographical and ecological metadata, and the literature reference from which these data were obtained. All the above data incorporated on ToxGen is available for its users and can be quickly accessed through searches within the “Taxon occurence/Living specimen” core module (File S1). The core information of the database is comprised of the downloadable Tri12 sequences with all available metadata. Although there are possibilities to import GenBank sequences for third-party annotation in semi-automated ways, in our case we decided to create a new dataset incorporating metadata from various sources. In addition, all the data stored in PlutoF are available through ToxGen GitHub repository (https://github.com/tomaszkulik/ToxGen) in both FASTA and CSV file formats. It allows all interested users to either manually download or harvest in automated ways, and use for local analyses. The downloadable data could be set up as a local reference sequence database and contribute to researchers using the next-generation sequencing analysis pipelines.

Each deposited sequence is indicated by its original GenBank accession number and is determined as one of the three (3ADON, 15ADON and NIV) genotypes identified in this study. The sequence analysis module of PlutoF was originally optimized for the identification and analysis of environmental ITS sequences of fungi, but has now been modified to enable direct identification of Tri12 genotypes. The workflow of such analysis (File S2) requires a blasting input sequences against the ToxGen database through the PlutoF seriateBLAST online tool (https://plutof.ut.ee/#/analysis/add).

Importantly, ToxGen allows holding sequences from various marker loci used for identification of fungi, such as: the partial translation elongation factor 1 − *α* (Tef1), two genes encoding the largest and second largest subunits of RNA polymerase (Rpb1 and Rpb2, respectively), internal transcribed spacer region (ITS rDNA), nuclear ribosomal RNA large subunit (28S or LSU rDNA) and mitochondrial small subunit (mtSSU rDNA). We aim to cover these loci in the future for the purpose of identification of Fusaria to the species level.

Although we have gone through a large number of peer-reviewed articles and websites to gather metadata on the reference strains, we cannot assume that all available metadata have been recorded in our database. PlutoF offers annotation capacities and storing new sequence data for all its registered users. Hence, contributions from the research community are welcome. Even minor additions of metadata or making corrections could be of significant importance for future studies on this important group of fungi.

### Comparison of ToxGen to other *Fusarium* databases

There are at least four sequence databases designed to provide reliable platforms for identification of Fusaria (Table 2). They hold sequences from multiple marker loci such as the Tef1, Rpb1 and Rpb2, calmodulin (Cam), *β*-tubulin (BenA), histone H3 (His), IGS rDNA, ITS rDNA, 28S rDNA and mtSSU rDNA. All of them can be used to explore the diversity of *Fusarium* and accurately identify new isolates based on their sequence similarity to previously characterized species (Geiser et al., 2004). The databases contain information associated with characterized strains, mainly providing a readily available reference to the current taxonomic statuses, as well as host and geographic records. ToxGen has many of the characteristics of other sequence databases (Table 2), but one of the major things that sets ToxGen apart from these are chemotaxonomic affiliations of the strains and annotations on their chemical diversity.

**Table 2:** Comparison of databases facilitating identification of toxigenic Fusaria.

Database	Identification through BLAST queries		Metadata				
	Species level	Tri genotype level	Taxonomic affiliations	Chemotaxonomic affiliations	Trichothecene production	Trichothecene- inducing host, substrate/media	Geographic and ecological metadata
Cyber-infrastructure for *Fusarium*^a^	+	−	+	−	−	−	−
Fusarium MLST DB^b^	+	−	+	−	+	−	+
GenBank^c^	+	−	+	−	−	−	−
MycoBank^d^	+	−	+	−	+	−	+
ToxGen^e^	−	+	+	+	+	+	+

**Notes.**

(+) available (−) unavailable

a http://www.fusariumdb.org/index.php.

b http://www.cbs.knaw.nl/fusarium/.

c www.ncbi.nlm.nih.gov.

d www.mycobank.org.

e https://plutof.ut.ee/.

A number of portals can be found on the Internet providing information on Fusaria to a broad audience including farmers, breeders, and scientists. Among the largest is US Wheat *&* Barley Scab Initiative (http://scabusa.org/). Other databases provide information of scientific interest. Most of them, such as Ensembl Fungi (http://ensemblgenomes.org/), MycoCosm (http://genome.jgi.doe.gov/programs/fungi/index.jsf, Grigoriev et al., 2014), *Fusarium graminearum* Genome Database (FGDB) (http://mips.gsf.de/genre/proj/fusarium/, Guldener et al., 2006), *F. graminearum* protein-protein interaction (FPPI) (http://comp-sysbio.org/fppi/, Zhao et al., 2009), FusariumSSRDB (http://webapp.cabgrid.res.in/ssr/home.html) integrate either fungal genomics or proteomics data and analytical tools for mycologists. More recently, a European Database of *Fusarium graminearum* and *F. culmorum* Trichothecene Genotypes was built aiming to characterize the trichothecene genotypes of strains from *Fusarium* species and to enhance the standardization of epidemiological data collection (http://catalogueeu.luxmcc.lu/, Pasquali et al., 2016a; Pasquali et al., 2016b). This database covers information on host plant, country of origin, sampling year and location, and previous crop of fungal isolates obtained from 17 European countries serving as a starting point for epidemiological analysis of potential spatial and temporal trichothecene genotype shifts in Europe. However, this database does not provide information on mycotoxin production by the fungi and lacks sequence data. To expand our ToxGen database, a joint collaborative effort has been set up between two databases, which will result in incorporation of data covering a large set of European field isolates.

## Discussion

The need for rapid and reliable diagnostic assays for mycotoxin producing Fusaria is imperative due to the high frequency of FHB, which leads worldwide to the contamination of grains with trichothecenes. Correct identification of Tri genotypes is elementary for all studies relating to population surveys, fungal ecology and mycotoxicology. For more than a decade the diagnostics of toxigenic *Fusarium* spp. experienced large methodological improvements through the development of molecular approaches for the identification and quantification of Tri genotypes (Pasquali & Migheli, 2014). However, these genotype specific assays are prone to either false positive or false negative results (Desjardins et al., 2008; Wang et al., 2008). It is rather easy to predict that the NGS technologies will have a tremendous effect on the diagnostics of mycotoxin producers. These high-throughput sequencing methods have been shown to outperform earlier molecular approaches in terms of resolution, and are currently taking over as the primary tool to assess fungal communities of plant-associated endophytes, pathogens, and mycorrhizal symbionts (Lindahl et al., 2013).

Even though molecular data are now widely used in fungal diagnostics, the identification of substantial amounts of fungi through sequence comparison is often hampered by the lack of well curated reference sequences, mostly due to difficulties related to culturing the strains (Begerow et al., 2010). Fortunately, Fusaria are easily isolated and grow on artificial media, which is reflected in the amount of strains maintained in culture collections. Notably, culture collections are experiencing continuous accumulation of fungal strains in their databases as a result of the growing number of *Fusarium*-related studies. The primary sources of information on the strains are scattered across the literature, which makes its proper utilization problematic, mainly due to: (i) multiple and inconsistent use of strain codes for individual strains, (ii) the use of incorrect *Fusarium* nomenclature, and finally (iii) inconsistent information published covering their mycotoxin profiles, chemotaxonomic affiliations, specifications of geographical and ecological data, etc.

Despite a common division into three major trichothecene B chemotypes/genotypes, fungal strains exhibit an enormous strain-dependent chemical diversity. Type B trichothecene producers display a high variation in levels of toxin production and are able to produce multiple toxins characteristic for other chemotypes (Mugrabi de Kuppler et al., 2011). Mixtures of multiple mycotoxins prove to have larger, synergistic effects than single compounds (Hou et al., 2013). Finally, some strains are able to produce high or even the relatively highest levels of mycotoxins not characteristic for their Tri genotypes (Castañares et al., 2014). This requires providing a wide spectrum of information covering molecular and chemical data, together with specific conditions inducing mycotoxin production, such as information on type of substrate, host and location. We believe that our new continuously updated database linking of sequencebased identification with chemical, as well as other metadata provides an ideal starting point for further studies.

The evolutionary history of the core Tri cluster is different from the history predicted by the species phylogeny, and it has been demonstrated that the polymorphism of some genes within this cluster is correlated with chemotypes within a panel of at least 22 species known to produce type B trichothecenes (Ward et al., 2002). Although other genes such as Tri3 and Tri13 were used as targets for previous genotyping assays, they appear to be inadequate for genotype identification through sequence comparison. The sequence of the Tri3 gene has been found not to be correlated with chemotypes in *F. aethiopicum* (O’Donnell et al., 2008), while the Tri13 gene contains large deletions/insertions (INDELs) which could result in errors with INDEL variant calling, driven by library preparation and sequencing biases.

In this study, we confirmed the usefulness of the Tri12 gene for the identification of trichothecene genotypes through sequence comparison. The polymorphisms in the Tri12 gene seem to be highly correlated with chemotypes, and display higher inter-genotype than intra-genotype variation. The usability of the Tri12 gene for Tri genotype identification has been confirmed in this study based on different analyses used for barcode evaluation. The sequence data of the Tri12 gene could serve as a target for the barcoding of Tri genotypes. Especially, sequence polymorphism at both edges of this gene appears to be highly correlated to chemotypes, which makes these regions highly relevant for the design of universal primers. We underline that our new database provides a highly relevant source of data for potential barcoding approaches by offering quick access to an informatively annotated set of reference sequences. The present initiative is the first step in covering the wide spectrum of mycotoxigenic fungi, and needs regular improvements.

It could be hypothesized whether reliable characterization of toxigenic fungi would require incorporation of more marker loci determining chemical diversity of Fusaria. Indeed, quite recently new fungal geno/chemotypes (Varga et al., 2015) and atypical strains (Kulik et al., 2016) have been discovered within *F. graminearum* s.s., which underlines the urgent need for updating. Identification of these new geno/chemotypes through sequence comparison requires incorporation of additional locus; Tri1 gene, which has been shown to determine the production of new mycotoxins (Varga et al., 2015). We aim to cover Tri1 and other loci in the future.

We invite experts working in the fields of fungal taxonomy, toxicology and epidemiology to join the effort. We believe that the results of such collaboration would be of great importance for all researches involved in studies on toxigenic fungi.

##  Supplemental Information

10.7717/peerj.2992/supp-1 File S1 Schematic presentation of workflow describing search of sequence and *Fusarium* metadata in the PlutoF platform

10.7717/peerj.2992/supp-2 File S2 Schematic presentation of workflow of the identification of Tri genotypes performed in the PlutoF platform

## Additional Information and Declarations

### DNA Deposition

The following information was supplied regarding the deposition of DNA sequences: GenBank Accession numbers:
KX197185, KX197186, KX197187, KX197188, KX197189, KX197190, KX197191, KT008433, KU572424.

### Data Availability

The following information was supplied regarding data availability: GitHub: https://github.com/tomaszkulik/ToxGen.
