# Supplementary material for: ToxGen: an improved reference database for the identification of type B-trichothecene genotypes in Fusarium
Source: PeerJ. 2017 Feb 15;5:e2992. doi: 10.7717/peerj.2992 (PMC5314956; doi:10.7717/peerj.2992)

PlutoF main page

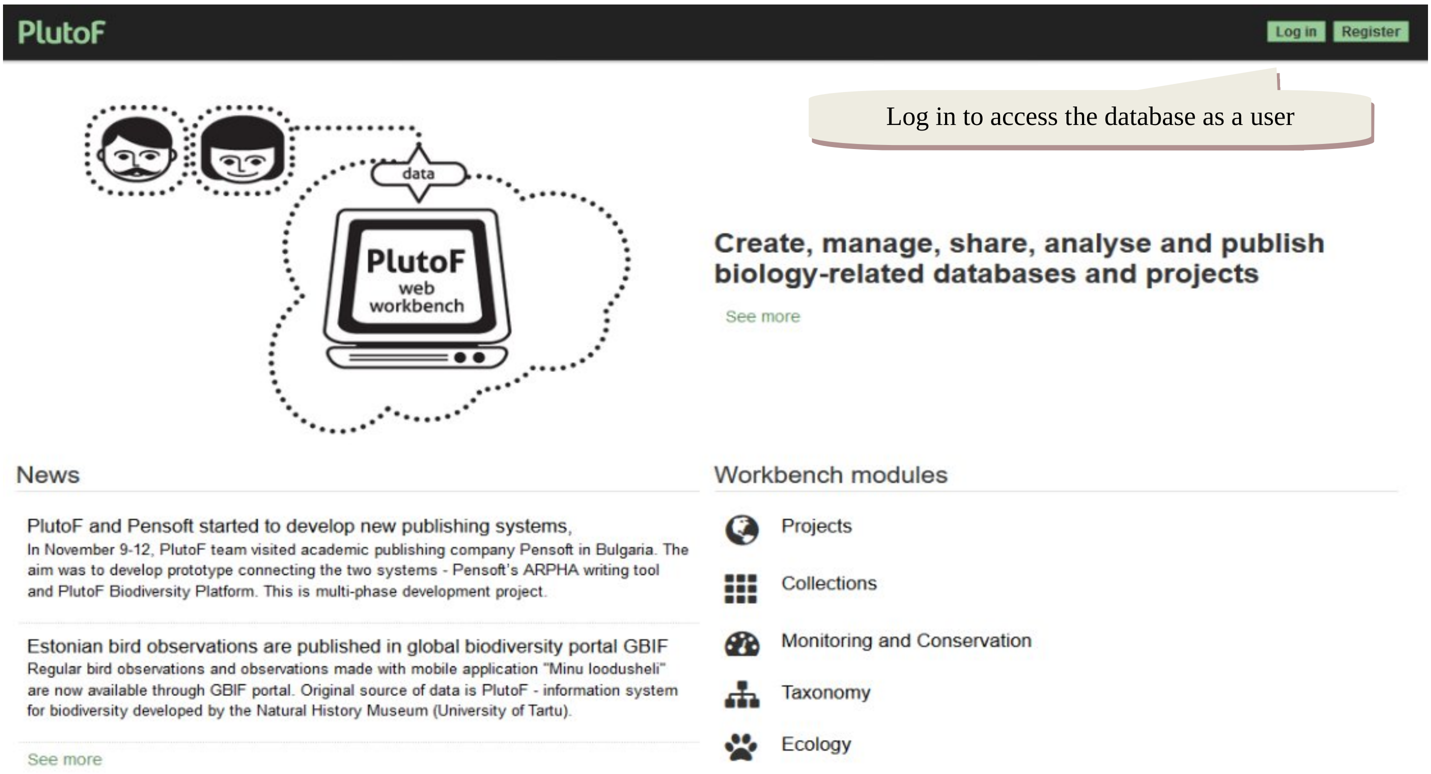

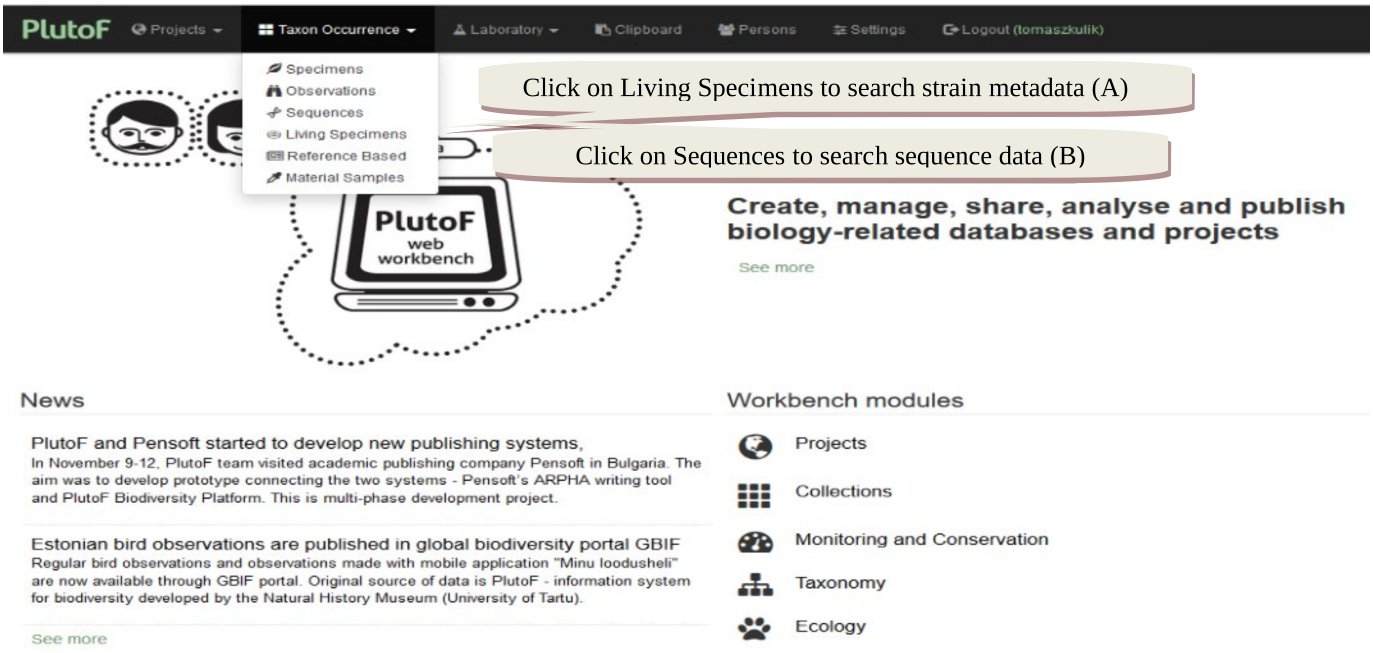

Searching for strain metadata (A)

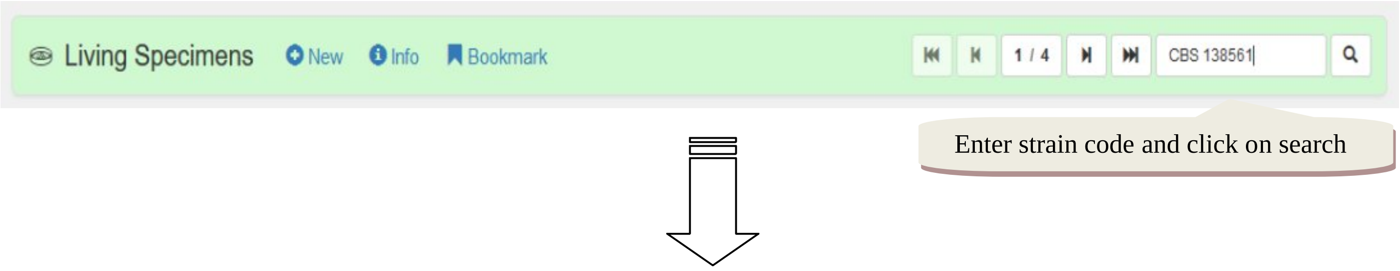

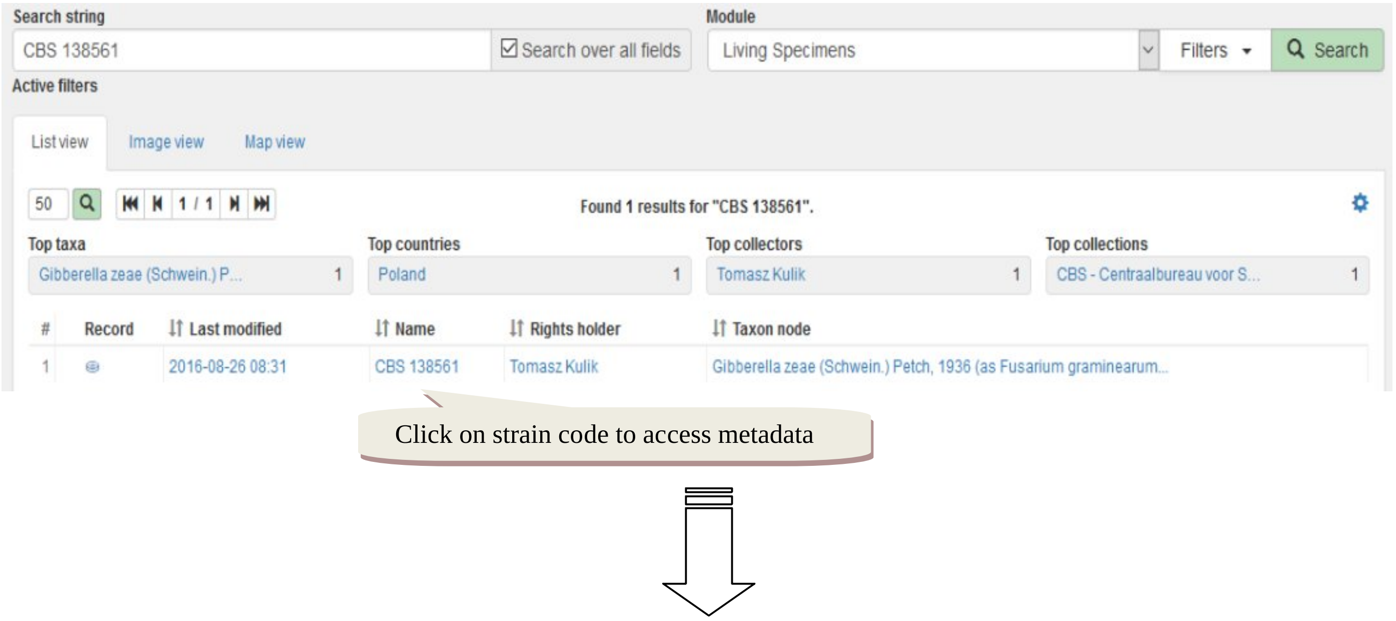

Accessing metadata

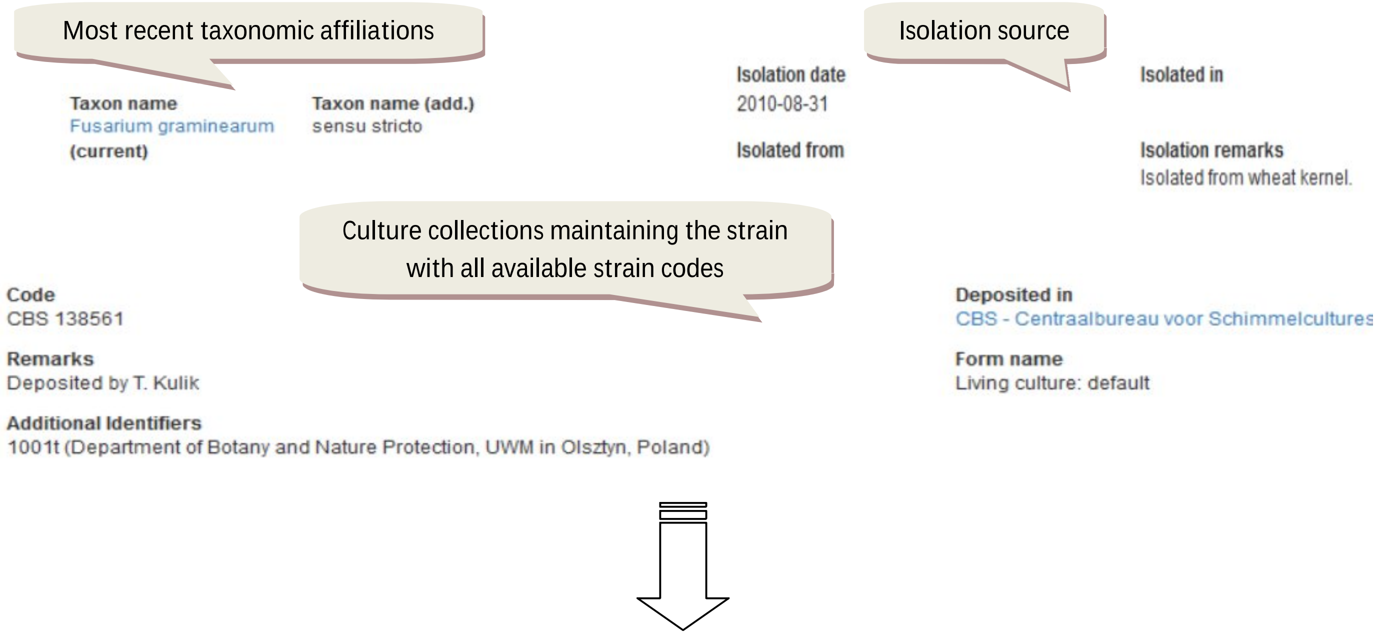

Map

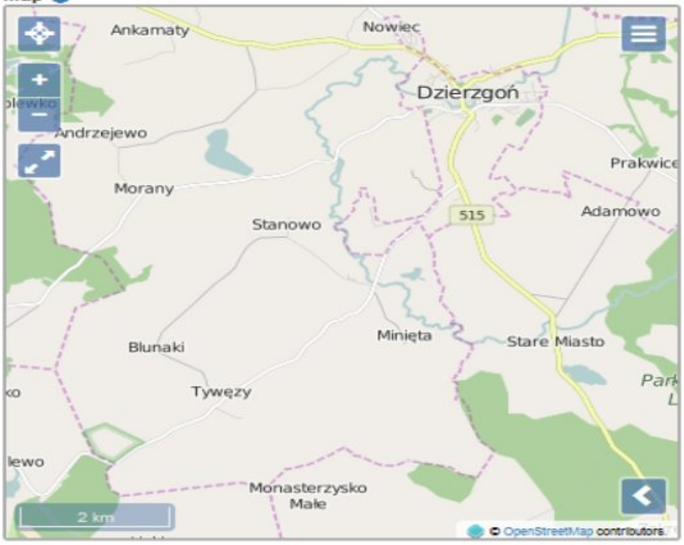

Sampling Area

Name
Dzierzgoń, Tywęzy

Country
Poland

Latitude

Longitude

State

District

Commune/City
Dzierzgoń

Locality text
Tywęzy

Accuracy (m)

Method

Elevation (min) (m)

Elevation (max) (m)

Depth (min) (m)

Depth (max) (m)

Sampling Event

Timespan begin

Timespan end

Event description

Geographic origin

Chemotype determination

Chemotype
15ADON

Genotype determination

Tri12 genotype
15ADON

Mycotoxin production

Substrate/medium remarks
The strain produced DON (2.16± 0.94 mg/kg), 3ADON (1.6± 0.3 mg/kg), 15ADON (1.42± 0.48 mg/kg), NIV (0.12± 0.02 mg/kg) and 4ANIV (0.96± 0.01 mg/kg) on YES medium

Searching for sequence data (B)

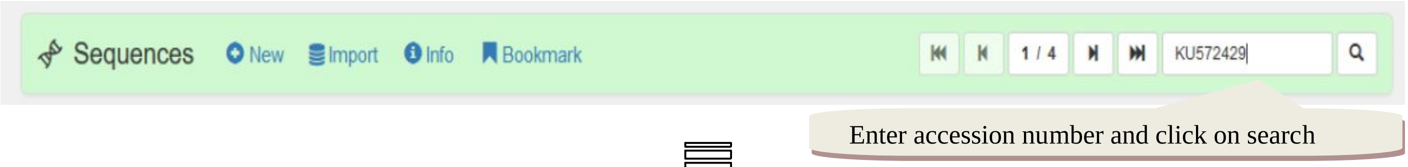

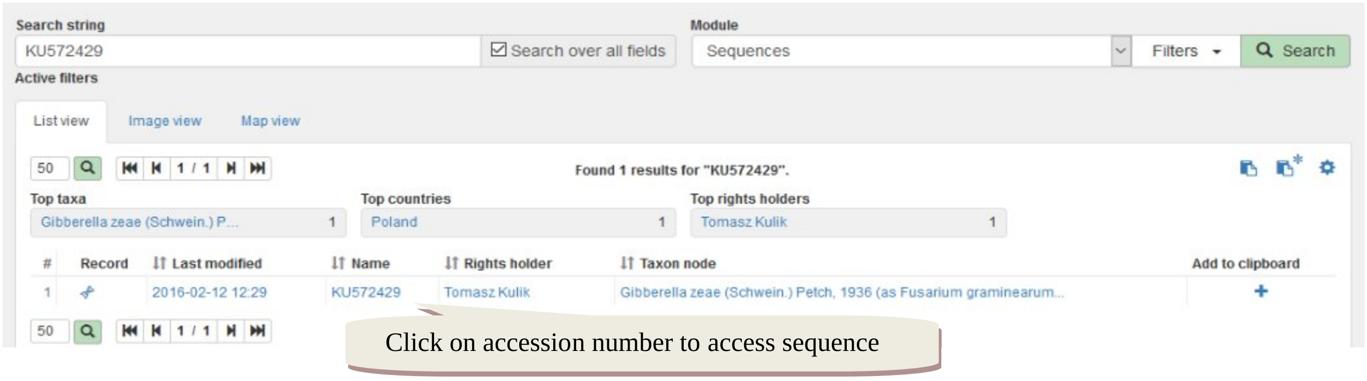

Accessing sequence data

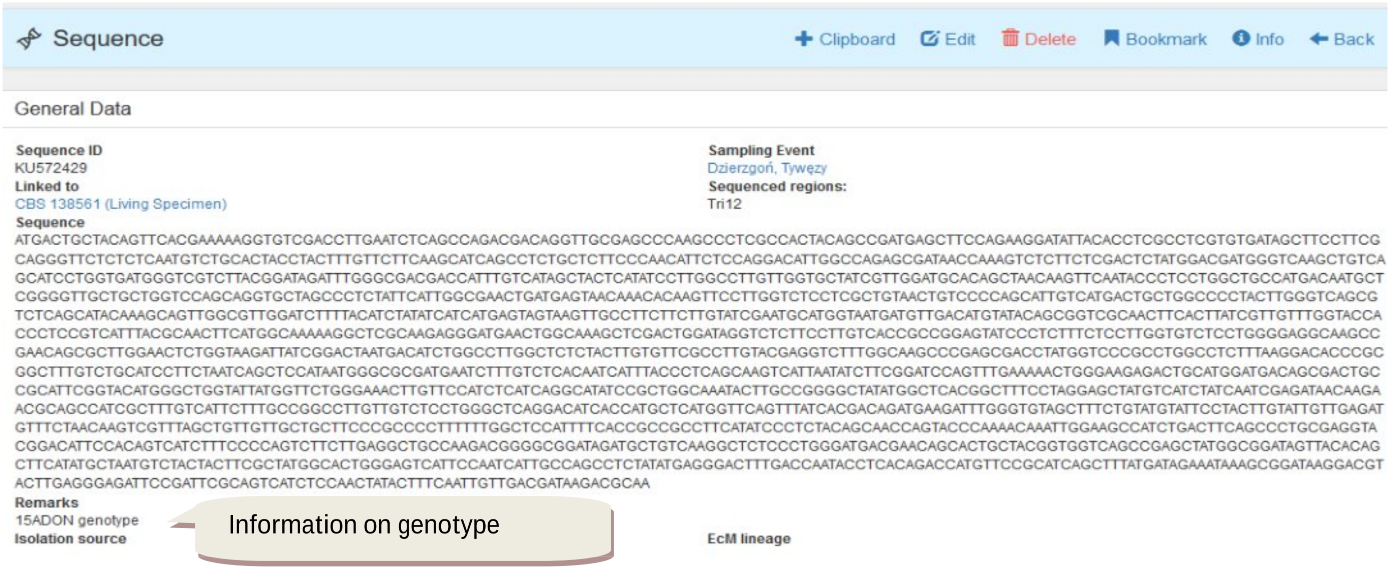

Supplement: File S1 [file peerj-05-2992-s001.pdf]
