# Supplementary material for: ToxGen: an improved reference database for the identification of type B-trichothecene genotypes in Fusarium
Source: PeerJ. 2017 Feb 15;5:e2992. doi: 10.7717/peerj.2992 (PMC5314956; doi:10.7717/peerj.2992)
Supplement: File S2 [file peerj-05-2992-s002.pdf]

Setting up BLAST search

General Data

Paste Tri12 sequences into BLAST input box

Analysis type
seriateBLAST

Name
Tri12 alignment

Choose input file in FASTA format
Type to find... Add new

Paste 1-1000 sequences in FASTA format

```
>
ATGACTGCTACAGTTCACGAAAAAGGTGTCGACCTTGAATCTCAGCCAGACGACAGGTTGCGAGCCCAAGCC
CTCGCCACTACAGCCGATGAGCTTCCAGAGGGATATTACACCTCGCCTCGTGTGATAGCTTCCTTCGCAGGG
TTCTCTCTCAATGTCTGCACTACCTACTTTGTTCTTCAAGCATCAGCCTCTGCTCTTCCCAACATTCTCCAGGA
CATTGGCCAGAGCGATAACCAAAGTCTCTTCTCGACTCTATGGACGATGGGTCAAGCTGTCAGCATCCTGGT
GATGGGTCGTCTTACGGATAGATTTGGGCGACGACCATTTGTCATAGCTACTCATATCCTTGGCCTTGTTGGT
```

Program Parameters

Select ToxGen

No of alignments
1 alignment shown

Output style
Condensed

Algorithm parameters
Similar sequences (fast)

☐ Unite ☐ GenBank ☐ Environmental Animal ☑ ToxGen

These are the finalized results of your BLAST run.
You can click here for a very condensed summary of the results

```
# BLASTN 2.2.26 [Sep-21-2011]
# Database: data/dbs4plutof/tri12
# Mode: ultra condensed output
Additional UNITE taxon name is in gray colour.

Query – The name of the query (input) sequence
Reference – The name of the reference sequence
Score – The BLAST score
E-value – The BLAST E-value
Prcnt – Percent identity over the BLAST alignment
MisM – The number of mismatches over the BLAST alignment
Qstart – The position in the query sequence where the BLAST alignment starts
Qend – [illegible]equence where the BLAST alignment ends
Rstart – [illegible]ence sequence where the BLAST alignment starts
Rend – [illegible]e sequence where the BLAST alignment ends
```

Click on the strain code to view strain metadata

Query 1 of [illegible]. 530092|AB060689||Gibberella_zeae_(as_Fusarium_graminearum)

Reference	Score	E-value	Prcnt	MisM	Qstart	Qend	Rstart
AB060689||Gibberella zeae (as Fusarium graminearum)	3520	0.0	100.00	0000	1	1906	1
AY102575||Fusarium asiaticum	3515	0.0	99.95	0001	1	1906	1
AY102604||Fusarium asiaticum	3487	0.0	99.69	0006	1	1906	1
KX197185||Fusarium	3290	0.0	97.85	0035	1	1906	1
KX197188||Fusarium louisianense	3256	0.0	97.54	0041	1	1906	1
KM408169||Fusarium ussurianum	3256	0.0	97.54	0041	1	1906	1
KU572426||Fusarium culmorum	3057	0.0	95.65	0074	1	1906	1
